# Sex differences in mobility recovery after hip fracture: a time series analysis

**DOI:** 10.3389/fpubh.2024.1434182

**Published:** 2024-08-27

**Authors:** Carl-Philipp Jansen, Monika Engdal, Raphael S. Peter, Jorunn L. Helbostad, Kristin Taraldsen, Beatrix Vereijken, Klaus Pfeiffer, Clemens Becker, Jochen Klenk

**Affiliations:** ^1^Department of Clinical Gerontology, Robert-Bosch-Hospital, Stuttgart, Germany; ^2^Geriatric Center, Heidelberg University Hospital, Heidelberg, Germany; ^3^Department of Neuromedicine and Movement Science, Norwegian University of Science and Technology, Trondheim, Norway; ^4^Institute of Epidemiology and Medical Biometry, Ulm University, Ulm, Germany; ^5^Department of Rehabilitation Science and Health Technology, Oslo Metropolitan University (OsloMet), Oslo, Norway; ^6^Study Center Stuttgart, IB University of Health and Social Sciences, Stuttgart, Germany

**Keywords:** sex differences, digital mobility outcomes, real-world walking, hip fracture, mobility

## Abstract

**Introduction:**

Sex differences are commonly reported for hip fracture incidence rates and recovery. Current knowledge about mobility recovery after hip fracture involves clinical assessments of physical capacity or patient-reported outcomes. Information on mobility performance during daily life is missing but relevant to evaluate patients’ recovery. Hence, it remains unclear whether sex differences exist in the longitudinal progression of mobility performance in hip fracture patients. To investigate this, we pooled data from four studies in Germany and Norway.

**Methods:**

In all studies, real-world mobility was assessed continuously over 1 to 7 days using a sensor fixed to the unaffected frontal thigh. All studies assessed mobility at different time points that were allocated to three distinct phases: Acute and post-acute phase (week 1–6), extended recovery (7–26), and long-term recovery (27–52). Sex-specific continuous trajectories of the median (50th percentile) as well as the 1st (25th percentile) and 3rd quartile (75th percentile) were estimated using quantile regression models with splines for daily walking and standing duration; number of sit-to-stand-to-walk transfers and walking bouts; mean walking bout duration; maximum number of steps per walking bout.

**Results:**

There were 5,900 valid observation days from *n* = 717 participants (mean age = 83.4 years, SD 6.1). The majority was female (75.3%), with similar sex distribution across all studies. Demographics of both sexes were comparable, but a higher percentage of women was living alone (69.0% compared to 40.9% in men) and had experienced an indoor fall leading to the fracture (74.3% compared to 67.4% in men). There were clear sex differences in mobility recovery. Women improved their mobility faster than men, but men showed larger increases later in the year after surgery. At the end of the first year, both sexes reached comparable levels in almost all mobility parameters.

**Conclusion:**

We identified varying aspects of mobility recovery between men and women, i.e., timely development of mobility recovery shows different patterns. Our findings support the consideration of sex differences in planning and implementing rehabilitation measures for hip fracture patients and highlight the need to provide adapted support at different time points. The underlying mechanisms of these sex differences need further investigation.

## Introduction

Hip fractures pose a major health issue worldwide (1), with projections of up to 6 million hip fractures per year by 2050 (1, 2). Frequent consequences of hip fractures are mobility limitations and reduced independence and daily functioning (3, 4). Mobility has been established as an important component of health and function; therefore, it is a major priority of hip fracture rehabilitation (5, 6). However, less than half of the people who sustain a hip fracture are able to recover to their previous mobility and function (7), making them vulnerable to low quality of life, functional dependence, high institutionalization rates, and increased mortality (8–11).

Hip fracture incidence rates in women are much higher than in men (12), and previous data suggests sex to be an important factor in recovery after hip fracture as well (13, 14). More specifically, sex differences have been reported for mortality (15), functional status (16), disability, gait speed, and depressive symptoms (17). Especially in factors in the spectrum of physical function, current results are based largely on data from patient-reported outcomes or supervised laboratory-based and clinical assessments of physical capacity. Through movement sensors, information on unsupervised free-living mobility in daily life can now be obtained in hip fracture patients with high ecological validity (18, 19). This provides information beyond snapshot views of single assessments, as well as continuous data on mobility *per se* and walking behaviour in particular. However, as shown in a recent literature review, this kind of assessment has hardly been utilized up to now (20), despite its high potential of providing deeper and more meaningful information on mobility recovery after hip fracture.

In a recent study, the progression of mobility in hip fracture patients within the first year after surgery was investigated in a broad manner, showing overall longitudinal trends in mobility in this patient group. For this, data from four studies in Germany and Norway were pooled in which movement sensors were applied at several time points (21). The present study is a secondary analysis of this data to investigate sex differences in the longitudinal progression of real-world mobility, operationalized as daily walking and standing duration, number of sit-to-stand-to-walk transfers and walking bouts, as well as mean and maximum values of walking bout duration. This set of digital mobility outcomes (DMOs) allows a broad view on the recovery of mobility patterns and helps to identify aspects that are different between men and women. Ultimately, this may help to take sex into consideration when personalizing rehabilitation.

## Materials and methods

### Study design and population

Due to the lack of sufficient prospective data during the first year after hip fracture surgery, the following four previously conducted randomized controlled trials were pooled to estimate the progression of mobility: the Trondheim Hip Fracture Trial (22) and the Eva Hip Trial (23, 24) conducted in Trondheim, Norway and the PROFinD 1 (25) and the PROFinD 2 (26) conducted in Stuttgart and Heidelberg, Germany.

The Trondheim Hip Fracture Trial compared comprehensive geriatric care in an orthogeriatric unit with standard orthopedic treatment, with the main outcomes being mortality, functional outcomes, nursing home residence, and (cost-)effectiveness. From 04/2008 to 12/2010, 397 community-dwelling older adults (≥70 years) were included in the study (22). The EVA-Hip Trial was a home-based supervised exercise program compared with usual care and was performed 4 to 6 months post-surgery. Outcomes were physical function and activity as well as cost-effectiveness. From 02/2011 to 03/2014, 143 community-dwelling older adults (≥70 years) were included (24). In PROFinD 1 the step-by-step rehabilitation protocol was compared with standard in-patient rehabilitation, investigating whether physical activity and fall-related self-efficacy were increased in hip-and pelvic fracture patients with fear of falling (25). From 04/2011 to 12/2013, 111 community-dwelling adults (≥ 60 years) were included (27). Lastly, in PROFinD 2, 185 community-dwelling hip-and pelvic fracture patients (≥ 65 years) with cognitive impairment according to Mini-Mental State Examination (MMSE; scores between 17 and 26) were included from 07/2015 to 02/2018. It was investigated whether a multifactorial home program would benefit usual care in terms of physical activity and functional performance (26).

All studies obtained ethical approval prior to study start. Trondheim Hip Fracture Trial: ClinicalTrials.gov NCT00667914; approved by the Regional Committee for Ethics in Medical Research in Central Norway (REK4.2008.335). EVA-Hip Trial: ClinicalTrials.gov NCT 01379456; approved by the Regional Committee for Ethics in Medical Research in Central Norway (REK2010/3265-3). PROFinD 1 and PROFinD 2 both received ethical approval from ethical committees at the University of Stuttgart (113/2011BO2) as well as at the Universities of Tübingen (150/2015BO1) and Heidelberg (S-256/2015). All four studies were conducted in accordance with the Declaration of Helsinki. Either participants or proxies provided written informed consent.

### Descriptive measures

Demographic data and clinical characteristics were obtained at baseline, including age, sex, body mass index (BMI), living alone at admission, and preferred habitual gait speed obtained 4 or 6 months after the surgery by a 4-meter walk test. For the PROFinD 2 and Trondheim cohorts, cognitive function was assessed using the MMSE (range from 0 to 30; higher scores indicate better cognitive performance) (28). In PROFinD 1, the Short Orientation Memory Concentration test (SOMC; range from 0 to 28; lower scores indicate better cognitive performance) was used (29). Cognition was assessed 4 months post-surgery (Trondheim cohorts) and 3 weeks post-surgery (Stuttgart cohorts).

### Digital mobility outcomes

In all studies, real-world mobility was assessed with the activPAL™ (PAL Technologies Ltd., Glasgow, United Kingdom) sensor, an accelerometer-based tri-axial sensor fixed to the unaffected frontal thigh using adhesive, waterproof tape. In the Trondheim Hip Fracture Trial, an older single-axis version of the sensor was used. The manufacturer’s software default settings were used to program the sensors and to process the recorded data, i.e., upright events were established with a minimum length of 10 s. The software algorithms detect postures (sitting or lying, standing, and walking), number of steps, and sit-to-stand transitions. The DMOs derived from the activPAL™ activity monitors have been validated in a population of older adults with impaired mobility, including hip fracture, showing high accuracy in classifying positions and recognizing transitions from sitting to standing positions (100%). Step counts during walking were underestimated, especially at low gait speeds (≤0.47 m/s) (30).

Mobility was measured continuously over 24 h for one to seven consecutive days. All studies assessed mobility at three time points following hip fracture surgery. Collectively, this led to eight follow-up periods within the initial year post-surgery, some of which overlapped. In the Trondheim Hip Fracture Trial, mobility assessment took place on the fourth day following surgery while the patient was hospitalized, followed by a four-day monitoring at 4 and 12 months post-surgery (at home). In the Eva Hip Trial, four-day mobility assessments were scheduled at home at 4, 6, and 12 months post-surgery. In the PROFinD 1 Trial, mobility was measured for 1 day at 3 to 4 weeks, and again at 6 weeks post-surgery during the in-patient rehabilitation stay, and for 7 days 4 to 5 months post-surgery (at home). In the PROFinD 2 Trial, mobility was measured for 3 days, with scheduled assessments at 2 to 3 months, 6 to 7 months, and 9 to 10 months post-surgery (all at home).

The activPAL software provides an event-based data output, with activities being defined as sitting or lying, standing, or walking. Based on this, the following DMOs were calculated for each 24 h cycle: Total walking duration (minutes), total standing duration (minutes), number of walking bouts per day, and number of sit-to-stand-to-walk transfers per day. Additionally, the maximum number of steps for each walking bout and the mean walking bout duration was determined.

### Data analysis and statistics

The DMOs at all assessment time points across the four trials were merged into one database, providing a longitudinal dataset, continuously covering the entire observation period. Only days with complete 24 h monitoring were included in the analyses. This was additionally verified by visually inspecting barcodes showing measurements throughout 24 h. The number of valid days for each participant and time point varied between 1 and 7 days.

The post-fracture observation period was limited to 1 year. Within this year, three periods for mobility recovery were defined to facilitate the interpretation of the results: the acute and post-acute period (weeks 1 to 6), the extended recovery phase (weeks 7 to 26), and the long-term recovery phase (weeks 27 to 52). Continuous variables were summarized as means and standard deviations (SDs), and categorical variables were presented as proportions and frequencies. Sex-specific continuous trajectories of the median (50th percentile) as well as the 1st quartile (25th percentile), and 3rd quartile (75th percentile) were estimated using quantile regression models with splines for each considered DMO. To investigate a possible effect of the intervention allocation and the different studies on the results, quartile-specific characteristics were calculated for these strata in a supplemental analysis. Additionally, several population characteristics were calculated depending on quartiles of walking duration to see whether proportions of both sexes remain stable. This is included in Supplementary Table S1. All analyses were performed using R 4.2.2 (with package quantregGrowth 1.7.0).

## Results

We included 5,900 valid observation days from 717 participants with a mean age of 83.4 (SD 6.1) years. Sample characteristics are shown in Table 1. The majority was female (75.3%), with similar sex distribution across the four studies. Both sex groups were comparable in age, BMI, type of fracture, cognitive function, and gait speed. However, a higher percentage of women were living alone (69.0% compared to 40.9% in men) and had experienced an indoor fall leading to the fracture (74.3% compared to 67.4% in men).

**Table 1:** Characteristics of the study population.

	Available data	Total	Women	Men
*N* (%)	717	717 (100)	540 (75.3)	177 (24.7)
Cohort, *n* (%)	717			
Trondheim Hip Fracture Trial		367 (49.8)	269 (75.4)	88 (24.6)
Eva-Hip Trial		130 (18.1)	100 (76.9)	30 (23.1)
PROFinD 1 Trial		93 (13.0)	68 (73.1)	25 (26.9)
PROFinD 2 Trial		137 (19.1)	103 (75.2)	34 (24.8)
Age [years], mean (SD)	717	83.4 (6.11)	83.6 (6.08)	82.8 (6.18)
BMI [kg/m^2^], mean (SD)	427	23.7 (4.00)	23.4 (4.13)	24.9 (3.34)
Living alone at admission, *n* (%)	714	443 (62.0)	371 (69.0)	72 (40.9)
Indoor falls, *n* (%)	510	370 (72.5)	283 (74.3)	87 (67.4)
Type of fracture, *n* (%)	717			
FCF		390 (54.4)	293 (54.3)	97 (54.8)
PTFF		277 (38.6)	207 (38.3)	70 (39.5)
STFF		50 (7.0)	40 (7.4)	10 (5.6)
Cognitive function^a^				
MMSE (0–30), median (IQR)	576	24.5 (7)	24 (6)	25 (7)
SOMC (0–28), median (IQR)	93	2 (6)	2 (6)	2 (3)
Gait speed (preferred) [m/s], mean (SD)^b^	619	0.56 (0.22)	0.56 (0.22)	0.58 (0.24)

SD, standard deviation; BMI, body mass index; FCF, fracture of cervical femur; PTFF, pertrochanteric femoral fracture; STFF, subtrochanteric femoral fracture; MMSE, Mini-Mental State Examination (higher scores indicate better cognitive performance); SOMC, Short Memory Concentration Test (lower scores indicate better cognitive performance).

a At month 4 (3 weeks for PROFinD 1 & 2) post-surgery, SOMC was assessed within PROFinD 1, MMSE within the other Trials.

b 4-Meter walk gait speed from Short Physical Performance Battery at month 4 (month 6 for PROFinD 2 Trial) post-surgery.

An overview on all estimated values at the end of each of the three phases is presented in Supplementary Table S2.

### Acute and post-acute phase

**Figure 1:**
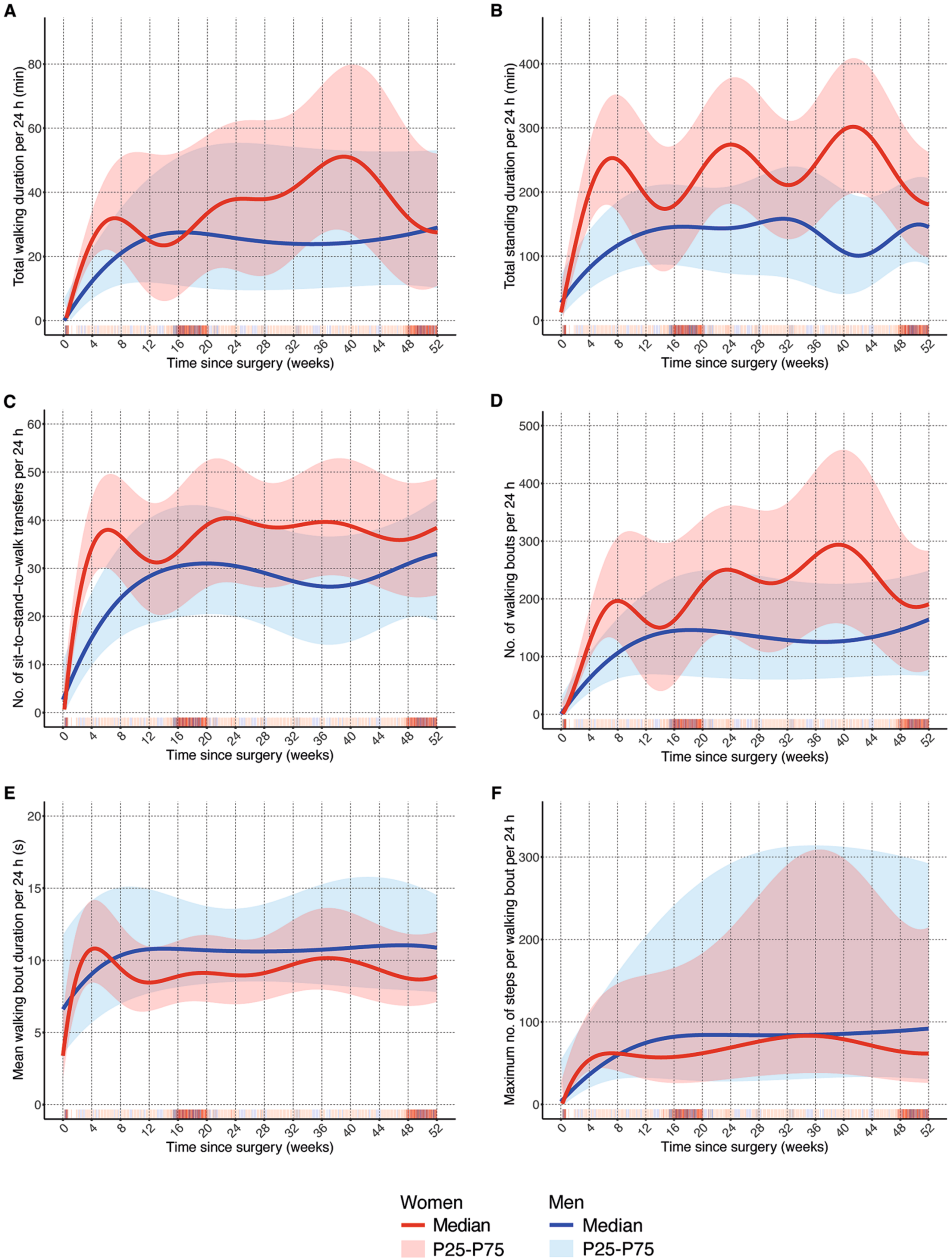
Comparison of one-year trajectories of mobility outcomes between men and women. **(A)** Total walking duration per 24 h in minutes; **(B)** total standing duration per 24 h in minutes; **(C)** number of sit-to-stand-to-walk transfers per 24 h; **(D)** number of walking bouts per 24 h; **(E)** mean walking bout duration per 24 h in seconds; **(F)** maximum number of steps per walking bout per 24 h; P25: 25th percentile; P75: 75th percentile.

Within the first 6 weeks, women had a steeper increase in mobility than men. This was the case for all parameters (Figures 1A–D), although both groups reached similar values after 6 weeks in walking bout duration (Figure 1E) and maximum number of steps per walking bout (Figure 1F). At week 6, women spent 31.1 (50th percentile; P50) minutes walking per day whereas men walked only 17.2 (P50) minutes per day (Figure 1A). The difference was more pronounced for daily standing, duration where women showed more than twice as much standing activity (245.8 min of standing per day vs. 101.3 min in men (P50); Figure 1B). The largest difference between sexes was found in the number of sit-to-stand-to-walk transfers (Figure 1C), where women reached their plateau at week six whereas men reached theirs around week 20. It is also important to note that women showed an increase to 38 transfers per day at week six, whereas men only had a median of 20 transfers at this time point. The number of walking bouts during the acute and post-acute phase was twice as high in women (179 walking bouts, P50) compared to men (86 walking bouts, P50; Figure 1D).

### Extended recovery phase

This phase between week 7 and week 26 was rather unsteady in women compared to men. After steep increases in mobility recovery during the acute and post-acute phase, women showed a clear drop until week 12 to 15 in total walking and standing duration, number of transfers and walking bouts, and walking bout duration (Figures 1A–E), but not in the maximum number of steps per walking bout (Figure 1E). Men, on the other hand, kept improving their mobility until week 12 to 15 and remained relatively stable from then onwards. There was a difference between volume parameters (Figures 1A–D) where women remained above the levels of men and overall, and frequency parameters (Figures 1E,F) where men showed higher values than women.

### Long-term recovery phase

Men remained relatively stable during the remainder of the year post-surgery (weeks 27 to 52). Towards the end of the year, men kept increasing their total walking and standing duration and the number of transfers and walking bouts (Figures 1A–D). In contrast, women showed steep decreases in these parameters from week 40 onwards (Figures 1A,B,D), although not in the number of transfers. Somewhat larger values for men in mean walking bout duration and the max. Number of steps per walking bout persisted until the end of the year (Figures 1E,F). Especially in the latter DMO, the range of the 25th to 75th percentile was large compared to the other parameters.

As is shown in Supplementary Table S1, the proportions of persons in each quartile remain stable in both sexes and show the same pattern for all characteristics included in this supplementary analysis.

## Discussion

In this paper, sex differences in real-world mobility were investigated, looking at a comprehensive set of DMOs in older adults after hip fracture. The key finding is that there are sex differences in mobility recovery. Women show steep increases in mobility parameters in the early phases, whereas men take more time to recover. At the end of the first year post-surgery, both sexes reached comparable levels in almost all investigated parameters. Looking at each parameter’s progression, the walking pattern of men is very different from women. Women tend to stand up and walk more often and for shorter durations than men, whereas men execute longer walks but stand up much less often. Reasons could be that women are required to take up on their “old roles” quite soon after their return to home whereas men may have a somewhat larger “grace period” in which they receive somewhat more support. Another reason could be that women perform more general activities in daily life whereas men have more specific occasions where they tend to be more active for longer periods of time. On another note, our data shows that a larger proportion of women is living alone compared to men, forcing them to take care of themselves upon their return to home whereas the majority of men have a spouse at home. Unfortunately, no data is available on the amount of support people may receive from caregivers in our sample.

Our findings differ from other studies that found better walking ability in men at four and 12 months after fracture (31). However, these differences disappeared upon age matching and were not related to real-world walking. Arinzon et al. also found better functional recovery after hip fracture in men than in women, but their study did not involve sensor-based real-world mobility and was based on a less generalisable sample than the sample in the present study (32). Unlike the aforementioned studies, no gender differences in functional independence after hip fracture were reported by Lieberman et al. (33), highlighting inconsistent evidence in this field. Results of the present study align with findings from Orwig et al. (17), who identified quicker recovery in gait speed and overall function of women in the early recovery stage at 2 months, followed by continuing recovery until plateauing at month six. Comparable to our findings, their study found that men caught up by month six and then continued to improve until 12 months, showing even higher walking capacity than women. Merging this evidence with our results, it seems that the progression of recovery comes with different timing between sexes, but the outcome at the end of the first year is comparable. This was previously found for laboratory-based capacity assessments in the aforementioned studies (17, 32) and is now supported by our findings on real-world performance as well.

There seems to be no obvious physiological reason why women should recover much faster. Factors that may explain the sex differences in recovery could be motivational issues, general behavioural patterns, and external factors in the way they address and handle their recovery. Previous research has shown that men were less motivated to be involved in health issues, although this finding was not based on hip fracture rehabilitation (34). Women are more active in the early phase, but reach a plateau after about 6 months. Motivation does not seem to be an issue initially, but at later stages, there either seems to be the notion of a sufficient recovery (in the sense that they can manage their key activities of daily living) and that no further improved is necessary, or movement habits have been formed that do not induce further improvement. Although the splines in Figures 1B,D show strikingly curvy lines, which is related to this method of data smoothing, the overall trend of women reaching this plateau earlier than men seems stable. There are several practical implications for the rehabilitation process. In men, stronger support in the early rehabilitation process is needed, as it seems that during this early phase they do not have the same pace in recovery as women. Men may also be particularly responsive to prehabilitation interventions. Women on the other hand should receive support at the beginning of what we defined as long-term recovery phase starting after 6 months, since they reach a plateau at this point in time. One way to provide such support could be home-based rehabilitation, for example focusing on improvement in gait parameters and physical function (23, 35).

### Strengths and limitations

This study involved a large sample of more than 700 hip fracture patients, where 5,909 observation days were recorded using the same type of movement sensor. This makes it the largest dataset on digital mobility endpoints in hip fracture patients so far. The sample characteristics show that the distribution of men and women in the four studies involved was similar and that study samples are representative of hip fracture patients overall, indicating that the data can be expected to be externally valid. Acknowledging study limitations, it needs to be pointed out that the data comes from intervention trials; so there might be intervention effects embedded within the data set. There is large variance and spread in the mobility performance patterns that needs to be considered when interpreting findings. This also shows the clinical reality and diversity of patients who improve very slowly as opposed to those who have steep inclines in mobility performance, but may also be grounded in the fact that data comes from different study centers and care pathways (e.g., time of discharge; duration of rehabilitation) in the early phase after surgery. It has to be acknowledged that there are time periods with fewer data points, as indicated by the density of vertical bars in Figure 1. Here, the model becomes less accurate, which might have led to less stable lines and might explain some of the fluctuations, especially in women. We have analyzed the main progression of all observations; individual patient trajectories were not estimated. Finally, the types of implants used for surgery were not considered, but might have had an impact on mobility trajectories.

## Conclusion

With the current set of digital mobility outcomes, we were able to reveal sex differences in mobility patterns during the first year after hip fracture surgery. We identified varying aspects of mobility recovery between men and women, showing that the progression of their recovery follows differing patterns over time. During the acute and post-acute phase women had a steeper increase in mobility outcomes than men, but reached a plateau during the extended recovery phase, where men in turn showed further increase. In the long-term recovery phase, men had more favorable mobility outcomes than women. Considering these sex differences in planning and implementing rehabilitation measures for hip fracture patients likely holds unexploited potential, but the underlying reasons for the observed differences need further investigation.

## Data Availability

The raw data supporting the conclusions of this article will be made available upon reasonable request by the authors, without undue reservation.
