# Supplementary material for: Sex differences in mobility recovery after hip fracture: a time series analysis
Source: Front Public Health. 2024 Aug 27;12:1434182. doi: 10.3389/fpubh.2024.1434182 (PMC11390126; doi:10.3389/fpubh.2024.1434182)
Supplement: Supplementary file 1 [file Table_1.DOCX]

Supplement 1: Characteristics of measurements stratified by quartiles of walking duration

|  |  |  |  | **Women (% of all measurements)** | | | |  |  | **Men (% of all measurements)** | | | |
| --- | --- | --- | --- | --- | --- | --- | --- | --- | --- | --- | --- | --- | --- |
|  | **All** |  | **Women** | **Q1** | **Q2** | **Q3** | **Q4** |  | **Men** | **Q1** | **Q2** | **Q3** | **Q4** |
| N (%) | 717 (100) |  | 540 (75.3) |  |  |  |  |  | 177 (24.7) |  |  |  |  |
| Number of measurements | 5,900 |  | 4,426 | 1,079 | 1,135 | 1,114 | 1,098 |  | 1,474 | 358 | 380 | 368 | 368 |
| Cohort, n (%) |  |  |  |  |  |  |  |  |  |  |  |  |  |
| Trondheim Hip Fracture Trial | 367 (49.8) |  | 269 (75.4) | 46.2 | 51.9 | 47.8 | 41.8 |  | 88 (24.6) | 41.9 | 47.1 | 45.1 | 43.8 |
| Eva-Hip Trial | 130 (18.1) |  | 100 (76.9) | 24.4 | 23.1 | 23.2 | 23.8 |  | 30 (23.1) | 30.4 | 17.1 | 22.6 | 26.6 |
| PROFinD 1 Trial | 93 (13.0) |  | 68 (73.1) | 12.3 | 8.6 | 10.7 | 14.0 |  | 25 (26.9) | 10.9 | 17.9 | 12.2 | 13.6 |
| PROFinD 2 Trial | 137 (19.1) |  | 103 (75.2) | 17.1 | 16.4 | 18.3 | 20.4 |  | 34 (24.8) | 16.8 | 17.9 | 20.1 | 16.0 |
| Age [years], mean (SD) | 83.4 (6.11) |  | 83.6 (6.08) | 85.2 | 84.6 | 83.1 | 81.5 |  | 82.8 (6.18) | 83.2 | 83.0 | 82.8 | 80.4 |
| BMI [kg/m^2^], mean (SD) | 23.7 (4.00) |  | 23.4 (4.13) | 22.7 | 23.6 | 23.7 | 23.6 |  | 24.9 (3.34) | 24.8 | 24.6 | 24.8 | 24.9 |
| Living alone at admission, n (%) | 443 (62.0) |  | 371 (69.0) | 72.1 | 73.6 | 64.7 | 64.8 |  | 72 (40.9) | 44.7 | 41.0 | 36.3 | 34.5 |
| Indoor falls, n (%) | 370 (72.5) |  | 283 (74.3) | 86.0 | 78.4 | 69.6 | 59.1 |  | 87 (67.4) | 89.1 | 76.8 | 64.8 | 39.9 |
| Type of fracture, n (%) |  |  |  |  |  |  |  |  |  |  |  |  |  |
| FCF | 390 (54.4) |  | 293 (54.3) | 47.9 | 52.1 | 61.1 | 61.2 |  | 97 (54.8) | 46.9 | 51.6 | 62.8 | 57.9 |
| PTFF | 277 (38.6) |  | 207 (38.3) | 45.5 | 39.4 | 32.6 | 32.3 |  | 70 (39.5) | 47.2 | 41.8 | 29.9 | 37.5 |
| STFF | 50 (7.0) |  | 40 (7.4) | 6.6 | 8.5 | 6.3 | 6.5 |  | 10 (5.6) | 5.9 | 6.6 | 7.3 | 4.6 |
|  |  |  |  | **Women (median of all measurements)** | | | |  |  | **Men (median of all measurements)** | | | |
| Cognitive function^#^ |  |  |  |  |  |  |  |  |  |  |  |  |  |
| MMSE (0-30), median (IQR) | 24.5 (7) |  | 24 (6) | 22 (18-26) | 24 (21-27) | 26 (23-28) | 26 (23-29) |  | 25 (7) | 24 (21-26) | 26 (20-28) | 28 (24-29) | 28 (26-30) |
| SOMC (0-28), median (IQR) | 2 (6) |  | 2 (6) | 4 (6) | 2 (7) | 4 (4) | 2 (4) |  | 2 (3) | 2 (2) | 2 (3) | 2 (6) | 4 (6) |
|  |  |  |  | **Women (mean of all measurements)** | | | |  |  | **Men (mean of all measurements)** | | | |
| Gait speed (preferred) [m/s], mean (SD)^$^ | 0.56 (0.22) |  | 0.56 (0.22) | 0.43 (0.18) | 0.52 (0.18) | 0.61 (0.19) | 0.68 (0.22) |  | 0.58 (0.24) | 0.44 (0.18) | 0.52 (0.16) | 0.62 (0.20) | 0.77 (0.27) |

SD: standard deviation, BMI: body mass index, FCF: fractura collum femoris, PTFF: pertrochanteric fractura femoris, STFF: subtrochanteric fractura femoris, MMSE: Mini-Mental State Examination (higher scores indicate better cognitive performance), SOMC: Short Memory Concentration Test (lower scores indicate better cognitive performance).

^#^ At month 4 (3 weeks for PROFinD 1 & 2) post-surgery, SOMC was assessed within PROFinD 1, MMSE within the other trails.

^$^ 4-Meter walk gait speed from Short Physical Performance Battery at month 4 (month 6 for PROFinD 2 Trail) post-surgery.

Supplement 2: Estimates of mobility parameters for selected time points (at the end of each of the three time points)

|  | **Week** | **P25** | **P50** | **P75** | **P90** |
| --- | --- | --- | --- | --- | --- |
| Total walking duration (min) |  |  |  |  |  |
| Women | 6 | 22.2 | 31.1 | 45.1 | 66.4 |
|  | 26 | 14.3 | 37.9 | 62.5 | 99.6 |
|  | 52 | 10.9 | 27.5 | 52.1 | 83.4 |
| Men | 6 | 9.6 | 17.2 | 29.1 | 44.5 |
|  | 26 | 9.5 | 25.1 | 55.3 | 87.0 |
|  | 52 | 10.2 | 29.0 | 53.1 | 83.7 |
| Maximum number of steps per bout |  |  |  |  |  |
| Women | 6 | 44 | 61 | 134 | 259 |
|  | 26 | 33 | 73 | 233 | 595 |
|  | 52 | 26 | 62 | 215 | 682 |
| Men | 6 | 27 | 49 | 135 | 324 |
|  | 26 | 28 | 84 | 300 | 719 |
|  | 52 | 31 | 92 | 292 | 814 |
| Number of sit-to-stand-to-walk transfers |  |  |  |  |  |
| Women | 6 | 30 | 38 | 49 | 60 |
|  | 26 | 26 | 40 | 49 | 60 |
|  | 52 | 24 | 38 | 49 | 60 |
| Men | 6 | 13 | 20 | 30 | 43 |
|  | 26 | 19 | 30 | 40 | 52 |
|  | 52 | 19 | 33 | 44 | 55 |
| Total standing duration (min) |  |  |  |  |  |
| Women | 6 | 179.6 | 245.8 | 335.8 | 424.1 |
|  | 26 | 173.2 | 265.1 | 373.6 | 470.7 |
|  | 52 | 98.0 | 180.7 | 262.3 | 344.0 |
| Men | 6 | 63.1 | 101.3 | 165.5 | 239.7 |
|  | 26 | 71.3 | 146.8 | 215.3 | 318.7 |
|  | 52 | 85.3 | 145.3 | 221.1 | 263.1 |
| Number of walking bouts |  |  |  |  |  |
| Women | 6 | 131 | 179 | 261 | 359 |
|  | 26 | 122 | 242 | 359 | 546 |
|  | 52 | 78 | 191 | 283 | 395 |
| Men | 6 | 53 | 86 | 138 | 202 |
|  | 26 | 61 | 138 | 244 | 350 |
|  | 52 | 67 | 164 | 249 | 326 |
| Mean walking bout duration (s) |  |  |  |  |  |
| Women | 6 | 8.0 | 10.4 | 13.7 | 16.3 |
|  | 26 | 7.1 | 9.0 | 11.4 | 14.8 |
|  | 52 | 7.1 | 8.9 | 12.0 | 15.8 |
| Men | 6 | 6.4 | 9.8 | 14.8 | 19.2 |
|  | 26 | 8.5 | 10.6 | 13.7 | 20.0 |
|  | 52 | 7.8 | 10.9 | 14.5 | 19.5 |

SD: standard deviation, BMI: body mass index, FCF: fractura collum femoris, PTFF: pertrochanteric fractura femoris, STFF: subtrochanteric fractura femoris, MMSE: Mini-Mental State Examination (higher scores indicate better cognitive performance), SOMC: Short Memory Concentration Test (lower scores indicate better cognitive performance).

^#^ At month 4 (3 weeks for PROFinD 1 & 2) post-surgery, SOMC was assessed within PROFinD 1, MMSE within the other trails.

^$^ 4-Meter walk gait speed from Short Physical Performance Battery at month 4 (month 6 for PROFinD 2 Trail) post-surgery.
